# Growth factor and co-receptor release by structural regulation of substrate metalloprotease accessibility

**DOI:** 10.1038/srep37464

**Published:** 2016-11-23

**Authors:** Liseth M. Parra, Monika Hartmann, Salome Schubach, Junzhi Ma, Peter Herrlich, Andreas Herrlich

**Affiliations:** 1 Leibniz Institute for Age Research, Fritz Lipmann Institute, Jena, Germany; 2 Washington University School of Medicine, Renal Division, St. Louis, MO, USA

## Abstract

Release of cytokines, growth factors and other life-essential molecules from precursors by a-disintegrin-and-metalloproteases (ADAMs) is regulated with high substrate-specificity. We hypothesized that this is achieved by cleavage-regulatory intracellular-domain (ICD)-modifications of the precursors. We show here that cleavage-stimuli-induced specific ICD-modifications cause structural substrate changes that enhance ectodomain sensitivity of neuregulin-1 (NRG1; epidermal-growth-factor) or CD44 (receptor-tyrosine-kinase (RTK) co-receptor) to chymotrypsin/trypsin or soluble ADAM. This inside-out signal transfer required substrate homodimerization and was prevented by cleavage-inhibitory ICD-mutations. In chimeras, regulation could be conferred to a foreign ectodomain, suggesting a common higher-order structure. We predict that substrate-specific protease-accessibility-regulation controls release of numerous ADAM substrates.

Release of many growth factors and cytokines as well as many receptors and adhesion molecules from precursor transmembrane proteins by metalloproteases (ectodomain cleavage) regulates numerous biological functions by release of important molecules involved in signal transfer between cells, or from the extracellular space to the inside of the cell^1^,^2^. The neuregulin precursor NRG1 and the hyaluronan receptor CD44 are such important examples of shed molecules. Neuregulin is important for neurite outgrowth and myelination as well as for heart development and function^3^,^4^. CD44 plays a dual role in growth regulation in that it mediates contact inhibition in association with hyaluronan^5^,^6^,^7^, yet it can also promote growth and metastasis of tumor cells^8^,^9^,^10^,^11^,^12^. Inappropriately upregulated or reduced ectodomain cleavage is associated with diseases (e.g. refs 13 and 14). Tight control of cleavage is therefore highly important for the organism.

Ectodomain cleavage is predominantly regulated by intracellular signaling pathways, mostly stimulated by G-protein-coupled-receptors and receptor-tyrosine-kinase (RTK) activation, involving protein-kinase-C (PKC) isoforms^15^,^16^,^17^. It is mainly carried out by one of two ADAM metalloproteases, ADAM10 or ADAM17 (reviewed in ref. 1). Yet, how ectodomain cleavage is regulated and made substrate-specific and how intracellular signals affect proteolysis on the cell surface remains unknown.

There is ample evidence that metalloproteases are regulated on several levels: transcription, trafficking, post-translational modification by pro-domain removal, dimerization, redox-regulated structural changes of its ectodomain, extracellular interaction with the metalloprotease inhibitor TIMP3 and C-terminal phosphorylation (reviewed in refs 1 and 18, 19, 20, 21, 22. Intracellular signaling can induce an open conformation of the ADAM17 catalytic domain as shown by studies with tight-binding ADAM17 inhibitors. However, surprisingly, the intracellular-domains (ICDs) of ADAM17 or ADAM10 can be removed without consequences for induced cleavage^22^,^23^,^24^. Thus, a clear molecular connection of how intracellular signaling influences the extracellularly located protease activity and cleavage has not been established thus far.

Ectodomain cleavage is also regulated on the substrate level, by ICD-modification of the substrate^15^,^16^, but what exactly these modifications regulate is unknown. NRG1 and CD44 are pre-associated with their ADAM enzymes in the absence or presence of cleavage stimuli/ICD-modifications, rendering proximity regulation of ADAM to its substrate unlikely (reviewed in refs 1 and 25). We thus asked whether cleavage-regulatory substrate ICD-modifications might turn the silent ADAM-substrate interaction productive by inducing structural substrate ectodomain changes that allow protease access.

Here we show that NRG1 and CD44 indeed undergo intracellular-signal-induced and ICD-modification-dependent structural changes of their ectodomains that allow ADAM protease access. PKCδ-induced ICD-phosphorylation regulates NRG1 protease accessibility, whereas CD44 requires induced ICD-dephosphorylation, which releases ICD-interaction with the tumor suppressor merlin (NF2). Our results identify a novel mechanism of regulated ectodomain cleavage likely to be relevant for numerous other ADAM substrates, including growth factors and cytokines.

## Results and Discussion

### Specific intracellular signal dependent substrate modifications induce conformational changes in the substrate’s ectodomain, resulting in increased protease accessibility

Because the ICD of ADAM10 and ADAM17 can be deleted without consequences for induced cleavage (see above and ref. 25), we hypothesized that specific substrate precursor protein ICD-modifications regulate ectodomain protease accessibility of the substrate.

**Figure 1:**
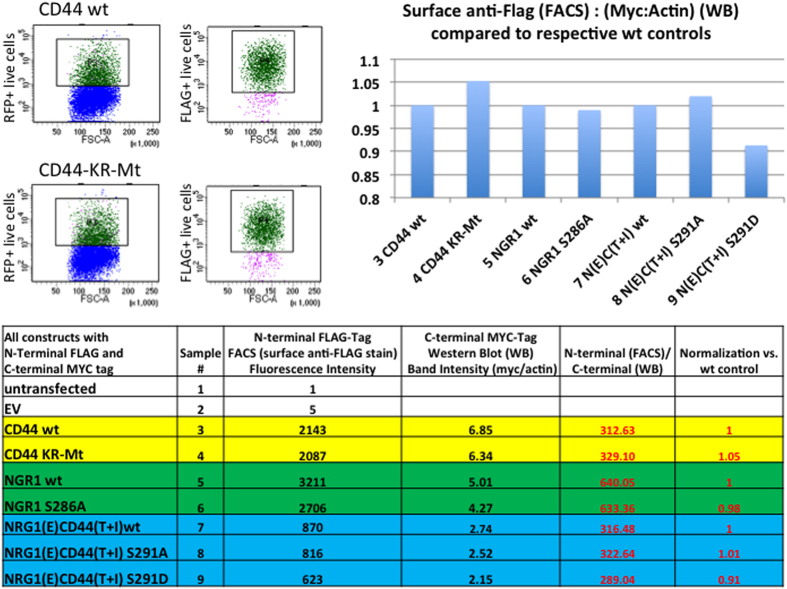
Cellular Expression and surface localization of CD44 and NRG1 constructs. HEK cells were co-transfected with a plasmid encoding RFP and one of the constructs listed. All these constructs carried an N-terminal FLAG tag and a C-terminal MyC tag. The cultures were incubated in the presence of ADAM and Gamma secretase inhibition. After 24 hours one aliquot of each culture was analyzed by FACS using anti-FLAG antibodies and Alexa-488-labelled secondary antibodies. For CD44wt and CD44KR-MT we show two representative FACS plots (upper left) and for the remainder of the constructs we show surface fluorescent intensity values as determined by FACS (Table column 3). These plots and values indicate the presence of the ectodomain of all studied constructs on the cell surface. Another aliquot of each culture was taken for lysis and Western blotting using either antibodies against the N-terminal FLAG or C-terminal MYC tag. The blots were quantified by Image J, the intensities normalized for actin. Western blot band intensities normalized for actin are shown in Table column 4 and normalization of these values to respective wt controls is shown in Table column 5 and also in the column diagram (upper right). Both MYC and FLAG data from the Western blots indicate proper expression of all studied constructs.

**Figure 2:**
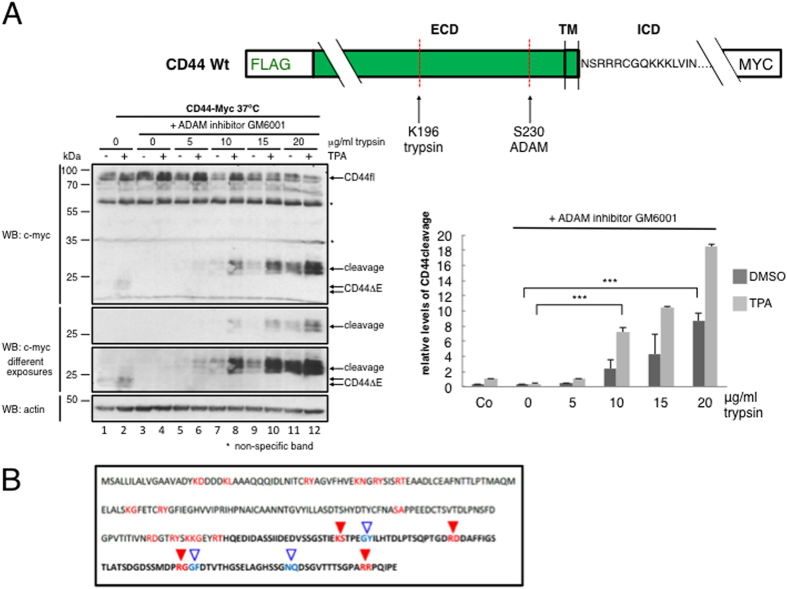
Intracellular signaling causes a change of ectodomain structure that determines protease accessibility of the CD44 ectodomain. **(A**) TPA treatment increases CD44 ectodomain trypsin sensitivity. (**B**) Schematic for putative trypsin sites hidden inside CD44’s three-dimensional structure. (**A**) Metalloprotease inhibition was used to block ADAM activity in CD44-transfected RPM-MC cells (GM6001 (15 μM)); 6 hours after TPA addition cells were probed with trypsin, lysed, and cleavage products resolved by SDS PAGE and immunoblot. γ-secretase inhibitor DAPT (5 μM) was added to all experiments. See sketch for trypsin cleavage site and tag location. Shown are one representative experiment of four independent experiments which have been evaluated by densitometry; the column diagram shows mean values of relative levels ±SD from four independent experiments.

**Figure 3:**
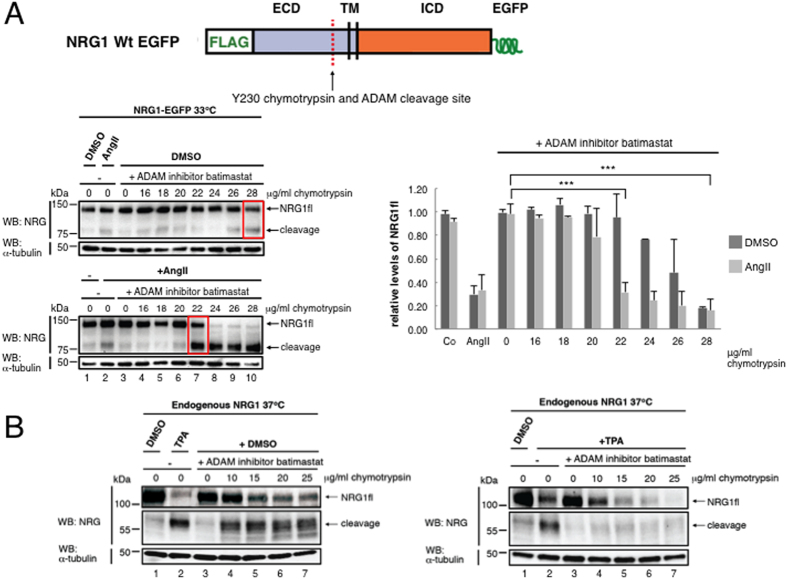
Intracellular signaling causes a change of ectodomain structure that determines protease accessibility of the NRG1 ectodomain. (**A**) Angiotensin II increases NRG1-EGFP ectodomain chymotrypsin sensitivity. Compare NRG1fl cleavage levels under DMSO (upper panel), or TPA stimulated conditions (lower panel); see red boxes. (**B**) TPA induced increased chymotrypsin sensitivity of endogenous NRG1 starting at the limiting concentration of 10 μg/ml of chymotrypsin. Compare NRG1fl levels under DMSO (left panel), or TPA stimulated conditions (right panel). The endogenous NRG1 cleavage fragment quickly degrades upon treatment with TPA and chymotrypsin (right panel). We did no use y-secretase inhibition in these particular experiments. (**A**) HEK cells stably expressing NRG1-EGFP were treated with the metalloprotease inhibitor batimastat (5 μM). 6 hours after TPA addition cells were probed with chymotrypsin, lysed, and cleavage products resolved by SDS PAGE and immunoblot. γ-secretase inhibitor DAPT (5 μM) was added to all experiments; BACE inhibitor (BACE-IV 1 μM) additionally in NRG1 experiments. See sketch for trypsin cleavage site and tag location. Shown are one representative experiment of four independent experiments which have been evaluated by densitometry; the column diagram shows mean values of relative levels ±SD from four independent experiments. (**B**) HEK cell expressing endogenous NRG1 only were treated as in (**A**). A further repeat experiment is shown in Supplemental Fig. 1.

To reveal such inducible structural changes, we probed the structural state of substrate ectodomains in presence or absence of cleavage stimuli, using accessibility to trypsin/chymotrypsin or soluble ADAM catalytic domain as a read-out. As substrates we used doubly-tagged molecules transfected into RPM-MC human pancreatic carcinoma cells (CD44) or HEK cells (NRG1). RPM-MC cells do not express CD44, permitting to examine overexpressed CD44 and its mutants without interference by *wt* endogenous counterparts. Both NRG1 and CD44 carried N-terminal FLAG tags; NRG1 and CD44 carried C-terminal c-myc tags or alternatively EGFP tags in select cases of NRG1 experiments (see schematics in Figs 2A, 3A and 6A). Surface expression of constructs was confirmed after transfection by FACS detection of the N′terminal FLAG ectodomain tag and by western blot detection of the N′terminal FLAG or C-terminal MYC tag (Fig. 1). For NRG1 there was no difference in surface expression whether a C-terminal c-myc or EGFP tag was used (data not shown). The surface expression shown here (Fig. 1) confirms results previously obtained by FACS analysis of transfected NRG1 expression contructs^16^. We tested regulated cleavage by endogenous ADAM for transfected CD44 or NRG1 and also for endogenous CD44 (Supplemental Fig. 1; MDA-MB-231 breast adenocarcinoma cells) and for endogenous NRG1 as indicated in the text below. Cleavage was induced by TPA or Angiotensin II (AngII, in HEK293T cells expressing the angiotensin-II-type1-receptor). For CD44 we chose trypsin because it produces only one cut close to the site of ADAM10 cleavage (schematic in Fig. 2A); other putative trypsin sites are apparently hidden inside CD44’s three-dimensional structure (schematic in Fig. 2B). For NRG1 we chose chymotrypsin. Chymotrypsin cuts NRG1 only once between F229 and Y230 (schematic in Fig. 3A)^26^. All putative ADAM17 cleavage sites reported are within the sequence ^226^MASFYKHLGIEFME^239^ surrounding the chymotrypsin site. The major cut *in vivo* is identical with that by chymotrypsin^27^. In these experiments, endogenous ADAM activity was blocked by batimastat (or GM6001), and γ-secretase was inhibited by DAPT, to exclude any other proteolysis (by γ-secretase and subsequent ICD processing) beyond the action of trypsin, chymotrypsin, or soluble ADAM.

**Figure 4:**
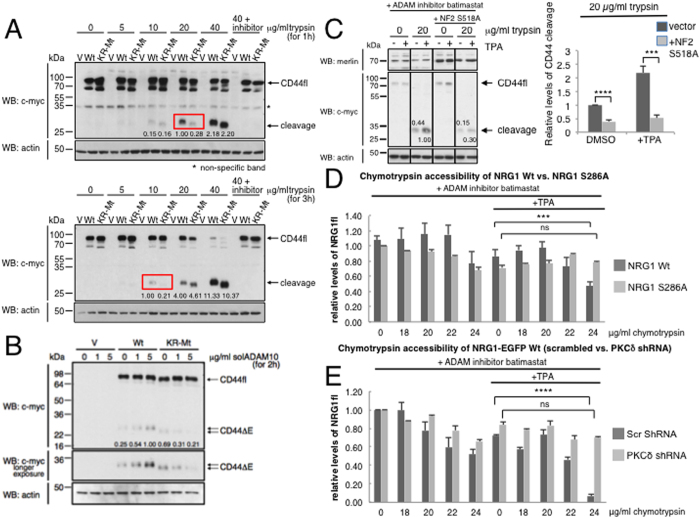
ICD modifications affect protease accessibility of the CD44 and NRG1 ectodomains. Ectodomains of CD44 *wt* or of uncleavable CD44 KR-Mt mutant show different protease accessibility to trypsin (**A**) or soluble ADAM10 (**B**). (**C**) Merlin regulates trypsin access to CD44 ectodomain. Constitutively active Merlin S518A blocks trypsin access to CD44 (right panel) when compared to *wt* merlin (left panel). (**D**) NRG1-S286A reduces NRG1 ectodomain accessibility to chymotrypsin. (**E**) PKCδ knockdown reduces NRG1 ectodomain accessibility to chymotrypsin. (**A**) CD44 *wt* and uncleavable KR-Mt were compared after exposure to increasing trypsin concentrations (37 °C) for 1 hr (upper) and 3 hrs (lower panel). Boxed pairs indicate concentration with largest differences between *wt* and KR-Mt. Conditions and cleavage detection as in Fig. 1. Relative levels of trypsin-cleaved CD44 shown as inserts. Based on size of single cleavage product, trypsin cuts in the ectodomain stalk region. 64 kDa band most likely represents under-glycosylated CD44. (**B**) Same as (**A**) but with soluble ADAM10 (37 °C; concentrations and times indicated). (**C**) RPM-MC cells were co-transfected with tagged CD44 *wt* and constitutively active merlin (NF2-S518A). CD44fl and C-terminal cleavage products (see “cleavage” middle panel) were detected as in Fig. 1. Merlin expression was detected by merlin antibody (upper panel). In (**C**) samples were run on the same gel but several lanes that showed other sample conditions using different trypsin concentrations were removed. (**D**) HEK293T cells were transfected with NRG1 *wt* or NRG1S286A, or co-transfected with NRG1 *wt* and PKCδ shRNA (**E**). Treatments as indicated. Chymotrypsin cleavage of full-length molecule was quantitated and normalized for tubulin (see column diagrams). Column diagrams show mean values of relative levels ± SD from three independent experiments; ns = non-significant, ***p-value < 0.001, ****p-value < 0.0001. Exemplary immunoblots see Supplemental Fig. 2A,B. Other statistical comparisons not shown: (3D) DMSO vs. TPA-treated NRG1wt: non-significant at 0, 18 and 20 μg/ml, but significant at 22 μg/ml (p-value 0.01) and 24 μg/ml chymotrypsin (p-value 0.0025). DMSO vs. TPA-treated NRG1S286A: non-significant. (3E) DMSO vs. TPA-treated NRG1wt plus scrambled shRNA: non-significant at 0, 18, 20 and 22 μg/ml, but significant at 24 μg/ml chymotrypsin (p-value 0.005). DMSO vs. TPA-treated NRG1wt plus PKCδ shRNA: non-significant.

The ectodomain of CD44 *wt* in TPA stimulated cells was indeed more sensitive to trypsin as compared to non-stimulated cells (Fig. 2A). While for non-stimulated cells 10 μg/ml trypsin generated the first faint trypsin cleavage product at a given temperature, TPA reduced the amount of trypsin required for the same effect to less than 5 μg/ml (Fig. 2A, compare lanes 7 and 8 with lanes 5 and 6). Further, in control stimulated cells at least 20 μg/ml of trypsin was required to reach a level of CD44 cleavage comparable to that detected after TPA stimulation and using only 10 μg/ml trypsin (Fig. 2A, compare lanes 8 and 11). Below we will show that these results can be reproduced using the soluble ADAM10 catalytic domain (see Fig. 4B).

NRG1 also became protease sensitive by cell stimulation with either TPA (not shown) or angiotensin II (AngII) (Fig. 3A). Full-length NRG1 (NRG1fl) was reduced upon AngII treatment to a level of 20% and a corresponding level of cleavage product was generated by 20–22 μg/ml chymotrypsin, a level reached by 28 μg/ml in non-stimulated cells (Fig. 3A, compare the square boxes in upper and lower panel, and the column diagram; a quantification of four independent experiments is shown in the column diagram). Correspondingly, similar induced changes of protease accessibility were detected for endogenously expressed NRG1 in HEK cells stimulated with TPA (Fig. 3B and Supplemental Fig. 2; note the difference in incubation temperature).

In summary, cleavage-regulatory signals alter substrate ectodomain structure as determined by a change in ectodomain accessibility to small-molecular-weight proteases.

### ICD modifications regulate protease accessibility of CD44 and NRG1 ectodomains

TPA or AngII cause posttranslational ICD-modifications of NRG1 and CD44 and ICD point mutations inhibit induced cleavage; e.g. in the binding domain of cleavage regulatory ERM/merlin proteins in CD44 (CD44-KR-Mt and CD44-S291D), or of NRG1-S286 or S289^16^. We used the same cleavage inhibitory ICD-mutations to test whether they prevented induced protease accessibility regulation. The ectodomain of CD44 *wt* was spontaneously more trypsin-sensitive than the non-cleavable CD44-KR-Mt mutant (Fig. 4A). The difference became particularly visible at a trypsin concentration of 20 μg/ml (incubation one hour) or 10 μg/ml (incubation three hours, see red squares and relative level quantifications within the immunoblots). CD44 *wt* and the mutant CD44-KR-Mt also differed in accessibility to soluble ADAM10 to a similar degree as when using trypsin. While 5 μg/ml of soluble ADAM10 catalytic domain cleaved CD44 *wt* in a 2-hour incubation, the mutant KR-MT was resistant to cleavage (Fig. 4B). Consistent with our results on mutation of CD44 S291 and its relevance for the binding of cleavage regulatory ERM/merlin proteins to the CD44 ICD^28^, protease accessibility of CD44 *wt* was inhibited by overexpression of a constitutively active merlin mutant (Fig. 4C; a quantification of three independent experiments is shown in the column diagram). The poorly cleaved ICD mutant NRG1S286A showed significantly reduced chymotrypsin cleavage at 24 μg/ml as compared to NRG1 *wt* (Fig. 4D; see exemplary immunoblot in Supplemental Fig. 3A). Downregulation of PKCδ which abolishes TPA- or AngII-induced NRG1S286 phosphorylation and inhibits NRG1 cleavage^16^ blocked TPA-induced protease accessibility of NRG1 almost completely (Fig. 4E; see exemplary immunoblot in Supplemental Fig. 3B).

**Figure 5:**
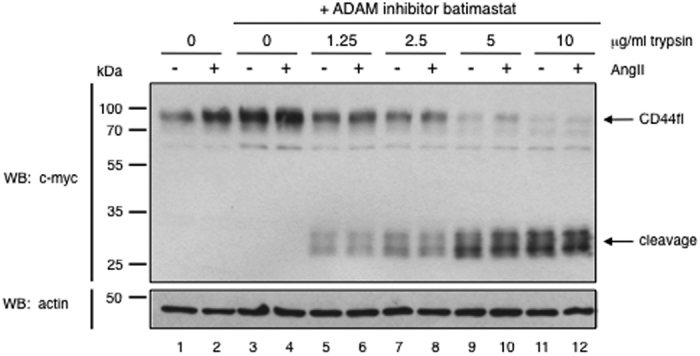
(**A**) Angiotensin II does not increase the trypsin accessibility of CD44. HEK293T cells stably overexpressing angiotensin II receptor 1 were transfected with tagged *wt* CD44, stimulated with 1 nM AngII for 2 hours, and trypsin cleavage was analyzed as in Fig. 2A.

The observed effects were indeed substrate and ICD specific: AngII, a stimulus that only induces cleavage of NRG1 (Fig. 3A, lower panel, compare lanes 1 and 2) but not of CD44 (Fig. 5, compare lanes 1 and 2), did not cause protease accessibility changes in CD44. Significant trypsin cleavage products where generated starting at 2.5 μg/ml, however, the CD44 cleavage products did not differ between control- and AngII-treated cells (Fig. 5, compare lanes 5 with 6, and 7 with 8). This result further supports our observation that specific cleavage of both substrates is addressed by different PKC isoforms^15^,^16^,^29^, namely PKCδ in the case of NRG1^16^ and another PKC isoform (not PKCδ) in case of CD44 as shown by inhibitor studies^29^.

In summary, the detected differences in ectodomain sensitivities of CD44 (trypsin), NRG1 (chymotrypsin) and of their respective mutants are highly suggestive of changes in ectodomain structure that occur in response to intracellular-signal-induced ICD-modifications and allow protease access at the metalloprotease site by either trypsin/chymotrypsin or soluble ADAM (tested for ADAM10, the physiological sheddase of CD44).

### Domain exchange between NRG1 and CD44: ICD modification regulates protease accessibility of the heterologous ectodomain

**Figure 6:**
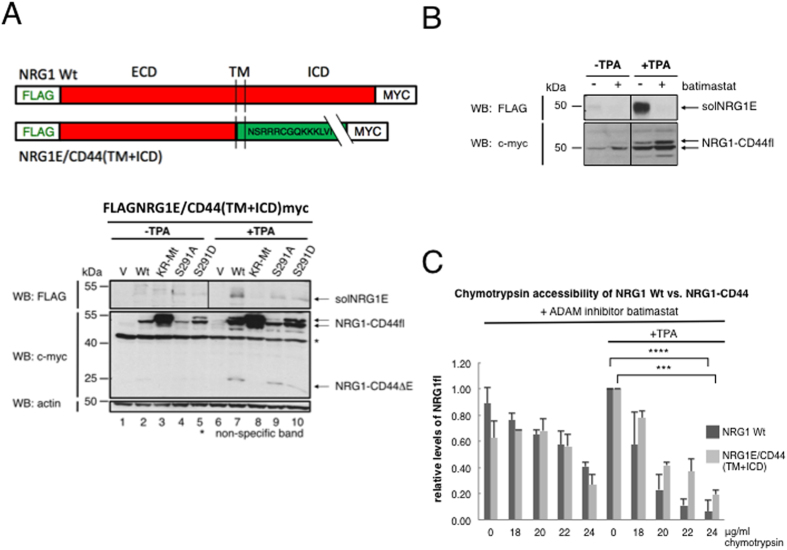
(**A**–**C**) ICDs modulate cleavage and protease accessibility of “foreign“ ectodomains in chimeras of CD44 and NRG1. (**A**) NRG1E/CD44(TM + ICD): CD44 ICD regulates cleavage of NRG1 ectodomain; effect of CD44 ICD mutants. See sketch for construction of chimeras. (**B**) Induced cleavage of NRG1E/CD44(TM + ICD) is inhibited by batimastat. (**C**) NRG1 wt and NRG1E/CD44(TM + ICD) show similar protease accessibility. (**A**,**B**) NRG *wt* and the NRG1E/CD44(TM + ICD) chimera were expressed in HEK293T cells E = ectodomain, TM = transmembrane domain, ICD = intracellular domain. The chimeric construct and its CD44 ICD mutants were transfected into RPM-MC cells and cleavage by TPA was analyzed as in Fig. 2. V = vector control. *Wt* = wild type. Batimastat (10 μM) was added 15 min prior to TPA. In (A) upper panel the solNRG1E samples were run on one gel, however an empty lane without sample that separated −TPA and +TPA was removed. The samples of the middle and lower panels were run on the same gel. The samples of (**B**) were also run on one and the same gel but several lanes that showed other samples were removed. (**C**) Protease accessibility regulation by TPA in the presence of batimastat (10 μM) was determined as in Fig. 1B. Column diagrams show mean values of relative level of ectodomain cleavage ± SD from three independent experiments.

NRG1 and CD44 ectodomains considerably differ in amino acid sequence but both undergo protease accessibility regulation suggesting similar secondary/tertiary structures. To test whether specific ICD-mediated protease accessibility regulation could be conferred to a “foreign” ectodomain, we performed ICD swap experiments between NRG1 and CD44 (see schematic in Fig. 6A). For CD44 we also constructed chimeric NRG1E(ectodomain)/CD44(TM + ICD) constructs with the relevant CD44 cleavage-regulatory ICD-mutations. The CD44 ICD in NRG1(E)/CD44(TM + ICD) indeed conferred TPA-dependent cleavage onto the “foreign” NRG1 ectodomain, as indicated by release of solNRG1E and of the C-terminal cleavage product NRG1-CD44ΔE (Fig. 6A, compare lanes 2 and 7). The CD44 cleavage-inhibitory ICD mutations KR-Mt as well as S291D, in contrast to the cleavage-activating S291A mutation, inhibited release of the “foreign” soluble NRG1 ectodomain (solNRG1E) (Fig. 6A, lanes 5 and 10) to a similar extent as it did for the “native” solCD44E in CD44 *wt*^29^; of note, expression levels, not loading, differed between mutant constructs for unknown reasons). The CD44E/NRG1ICD chimeras showed cleavage regulation just like NRG1 *wt* as determined by detection of an NRG1 specific inhibition profile with PKC blockers (data not shown). Cleavage could also still be blocked by the metalloprotease inhibitor batimastat (shown for NRG1/CD44 chimera in Fig. 6B). Finally, ICD-modifications also conferred protease accessibility regulation to a “foreign” ectodomain. As compared to control cells, TPA enhanced chymotrypsin cleavage of the NRG1E/CD44(TM + ICD) chimera almost as much as of NRG1 *wt* (above a chymotrypsin concentration of about 20 μg/ml) (Fig. 6C, compare to Fig. 3A). This observation is of particular significance for the validity of our novel model of ectodomain protease accessibility regulation.

These results suggest that accessibility of the substrate’s ectodomain is regulated by a higher-order structure. ADAM dependent cleavage occurs at defined cleavage sites (reviewed in refs 1, 30 and 31. Induced changes in ectodomain structure likely expose these sites and allow the protease catalytic cleft access to the ADAM cleavage site.

### “Signal transfer” through the plasma membrane requires substrate dimerization

**Figure 7:**
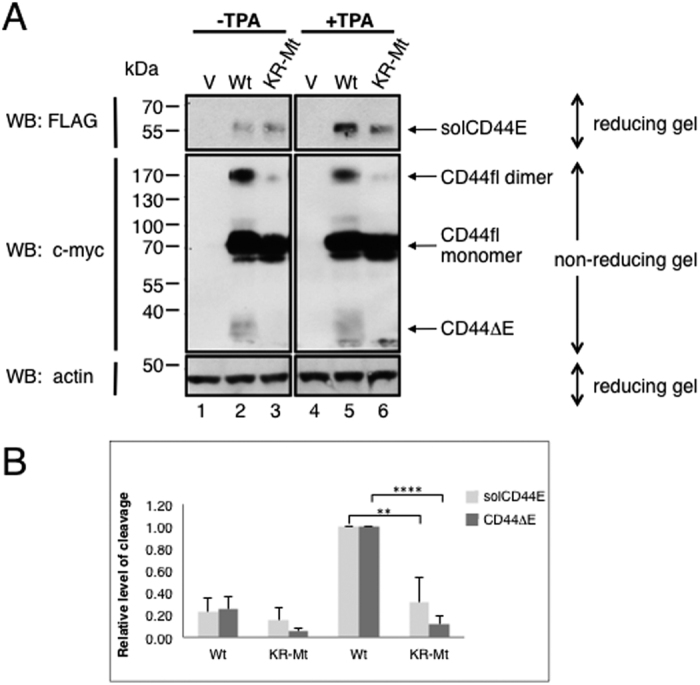
Preferential cleavage of CD44 homodimers. (**A**) CD44 dimers could be detected on denaturing SDS-PAGE gels under non-reducing conditions^25^. The uncleavable CD44 KR-MT mutant dimerizes only minimally. TPA did not influence dimerization but enhanced TPA-induced release of soluble CD44 ectodomain. CD44 wt or its KR-MT mutant were transfected into RPM-MC cells and cleavage by TPA was analyzed as in Fig. 2. (**A**) samples were run on one gel, but the empty lane between −TPA and +TPA was removed. (**B**) The column diagram shows mean values of relative levels of ectodomain cleavage ± SD obtained by resolution on reducing gels from three independent experiments.

Transmission of a conformational change through the plasma membrane cannot be achieved by single-pass transmembrane proteins. The intracellular protein kinase of RTKs for example is activated by ligand-induced relative positioning of individual subunits in receptor dimers in response to ligand binding (“outside-in” signaling) (reviewed in ref. 32).We hypothesized that “inside-out” signaling which confers substrate protease accessibility would also require dimerization. Both NRG1 and CD44 form homodimers which may represent an essential pre-condition for their regulated ectodomain cleavage^25^. Because CD44 dimers are stabilized by S-S bridges, part of CD44 migrated as dimer in a non-reducing gel. However, the un-cleavable CD44 mutant (KR-MT) did almost not dimerize at all (Fig. 7, lanes 2 and 3). TPA induced ectodomain cleavage products above a low basal level are detected in CD44 *wt* but only barely in case of the mutant KR-MT (Fig. 7, compare lanes 2 and 3 with lanes 5 and 6), correlating with trypsin accessibility reduction of CD44 KR-MT (Fig. 4A).

ADAM and substrate are already pre-associated in a silent manner in the absence of a cleavage stimulus^25^,^33^,^34^; e.g. Notch or CD44 with ADAM10, as well as NRG1 with ADAM17. Sites for interaction with certain select substrates within the so-called membrane proximal domain (and outside of the catalytic cleft) have been described for ADAM17^35^. In case of Notch, binding of the ligand permits protease action^36^, a structural modulation triggered directly through the ectodomain. Protease accessibility regulation of the substrate’s ectodomain by intracellular signaling requires a different mechanism. It is theoretically possible that ADAM-Substrate heterodimers are required for protease accessibility regulation. However, based on our results with chimeric constructs and deletion of the ADAM10 ICD, and based on the need for dimerization, we postulate ICD-modification-mediated positioning of one substrate dimer chain relative to the other in a homodimer (work presented here and in ref. 25). This might trigger the conformational change of higher order structure necessary to allow access of the catalytic cleft of the pre-associated ADAM protease to the substrate’s ADAM cleavage site.

Dimer dependent signal transfer across the cell membrane is not without precedence in both directions “inside-out” (as here) and “outside-in”. In addition to RTKs, integrin regulation speaks to this process. Binding of ECM ligands to integrin heterodimers causes ICD-modification^37^. Conversely, interaction of the integrin beta chain ICD with talin appears to disrupt the “silent state” of the heterodimer, initiating “inside-out” signaling^38^.

To understand precisely what happens to dimers during signal transfer requires crystallization of the two states, prior and after stimulation by ligand or, in our case, ICD-modification. Such experiments solved the activation mechanism of RTK homodimers or heterodimers. Ligand binding creates altered positioning of the two EGF receptor subunits across the membrane, activating their ICD protein kinase activity (reviewed in refs 32, 39 and 40). Such information is pending for ADAM substrates and their dimers.

In summary, as the major message of this paper, we show that silent interactions between metalloprotease and its substrates are converted into an active state by cleavage regulatory ICD-modifications that induce signal-transfer to the substrate’s ectodomain allowing protease accessibility, a process that requires substrate dimerization. We hypothesize that this novel cleavage regulatory mechanism extends to many ADAM substrates permitting the controlled and specific release of life-essential regulatory molecules such as growth factors, cytokines and decoy receptors. As such, this mechanism might be accessible to therapeutic intervention in order to inhibit the action of specific metalloprotease substrates.

## Experimental Procedures

### Reagents.

DNA-oligonucleotides [Metabion]; TPA, DAPT, Angiotensin II, Trypsin, Chymotrypsin, TCA-DOC [Sigma]; beta-secretase-(BACE)-inhibitor-I and batimastat (BB94) [Calbiochem]; GM6001, Compound E [Enzo]; soluble ADAM10 catalytic domain and soluble NRG1 [R&D systems]; DAPI [Vecta]. Lipofectamine 2000 [Invitrogen]; Fugene6, Complete protease inhibitor cocktail [Roche].

### Antibodies.

Anti-FLAG (M2 and SIG1-25) [Sigma]; ADAM10 (735–749) and ADAM17 (TACE) (807–823) [Calbiochem or R&D Systems]; another ADAM17 C-terminal antibody was a gift from Carl Blobel [Hospital for Special Surgery, New York]; c-Myc (9E10), HA (F-7), GFP (B-2), NF2 (merlin) (C-19; C-18; B-12), neuregulin-1α/β1/2 (C20), and Actin (I-19) [Santa Cruz Biotechnology]; PKC**δ** (D10E2) and GFP (4B10 and D5.1) [Cell Signaling Technology]; α-Tubulin (ab4074) [Abcam].

### Plasmids.

pcDNA3.1 [Invitrogen] based plasmids expressing cDNAs encoding CD44wt, NRG1wt or the merlin mutant (NF2 S518A and S518D)^7^,^28^. The sequence encoding the standard isoform of rat CD44 was subcloned into the NotI/XbaI sites of pFLAG-myc-CMV-21, to generate CD44 with N-terminal FLAG and C-terminal myc epitope. A retroviral construct encoding FLAG-pro-NRG1-EGFP has been described^15^. CD44 and NRG1 ICD mutants as well as ICD mutants of the chimeric constructs were generated by site-directed mutagenesis; NRG1S286A was as in ref. 16. NRGE/CD44(TM + ICD) was generated by subcloning the NRG1 ectodomain sequence into pFLAG-myc-CMV-21 containing CD44 such that the CD44 ectodomain was replaced. CD44E/NRG (TM + ICD) was generated in analogous fashion. All constructs were verified by sequencing.

### Cell Lines and Transfections.

NIH3T3 fibroblasts were from the European Collection of Animal Cell Cultures [Salisbury, UK]. The human melanoma RPM-MC cells, negative for CD44, were provided by Ivan Stamenkovic [University of Lausanne, Switzerland], mouse embryonic fibroblasts (MEFs) with *adam10* deletion by Paul Saftig [University of Kiel, Germany]. The stable HEK cell lines HEKNE WT and HEKNE NRG1S286A were created by retroviral infection with FLAG-NRG1β1a-EGFP (wt and S286A mutant)^16^. All cells were grown in DMEM supplemented with 10% FBS. DNA and siRNA transfections were performed in 6-well plates (protease accessibility studies) using Lipofectamine 2000.

### Inhibited Cleavage Conditions.

Metalloprotease activity was blocked by culturing cells with broad-spectrum hydroxamate-based metalloprotease inhibitors, 15 μM GM-6001 or 5 μM batimastat (BB94) at 15–30 min prior to TPA or AngII stimulation. In addition, γ-secretase activity was blocked by 5 μM DAPT or by 10 μM Compound E.

### Limited Proteolysis in Cultured Cells.

Cells were seeded in 6-well plates and incubated with different concentrations of trypsin for CD44 and chymotrypsin for NRG1 (in serum-free medium) or soluble recombinant ADAM10 (in assay buffer: 25 mM Tris pH 8.0, 2.5 μM ZnCl2, 0.005% w/v Brij-35). Proteolysis was stopped by the addition of 1x complete protease inhibitor cocktail. The trypsin and chymotrypsin cleavage sites were determined using PeptideCutter provided by the ExPASy Bioinformatics Resource Portal.

### Precipitation of Proteins by TCA-DOC (Trichloro Acetic Acid – Na Deoxycholate).

For detection of soluble CD44 ectodomain or neuregulin, cells were cultured in serum-free medium. Culture supernatants were pre-cleared at 10,000 rpm for 10 min, then mixed with 1/100 volume of 2% DOC, vortexed and kept on ice for 30 min. Then 1/10 volume of 100% TCA was added, and the samples were kept at 4 °C overnight. The precipitate was recovered by centrifugation at 15,000 g for 15 min, rinsed twice with acetone and re-dissolved in RIPA buffer (50 mM Tris-HCl pH 8.0, 150 mM NaCl, 1% NP-40, 0.5% sodium deoxycholate, 0.1% SDS).

### Statistical Analysis.

For statistical analysis the intensity of bands from immunoblots was quantified using ImageJ and Image Lab^®^ (Biorad, Hercules, CA) software. All values in column diagrams are reported as mean ± standard deviation (SD). Statistical analyses of experiments were performed using unpaired Student’s two-tail t test of data analyzed from at least three to four independent experiments.

## Additional Information

**How to cite this article**: Parra, L. M. *et al*. Growth factor and co-receptor release by structural regulation of substrate metalloprotease accessibility. *Sci. Rep.* **6**, 37464; doi: 10.1038/srep37464 (2016).

**Publisher's note:** Springer Nature remains neutral with regard to jurisdictional claims in published maps and institutional affiliations.

## Supplementary Material

Supplemental Figures and Legends
