# Supplementary material for: Growth factor and co-receptor release by structural regulation of substrate metalloprotease accessibility
Source: Sci Rep. 2016 Nov 23;6:37464. doi: 10.1038/srep37464 (PMC5120278; doi:10.1038/srep37464)
Supplement: Supplemental Figures and Legends [file srep37464-s1.pdf]

Growth factor and co-receptor release by structural regulation of substrate metalloprotease accessibility.

Liseth M Parra[1,2*], Monika Hartmann[1*], Salome Schubach[1], Junzhi Ma[1], Peter Herrlich[1] and Andreas Herrlich[2**]**

[1] Leibniz Institute for Age Research, Fritz Lipmann Institute, Jena, Germany

[2] Washington University School of Medicine, Renal Division, St. Louis, MO, USA

* shared first authorship, ** shared senior authorship

To whom correspondence should be addressed: Andreas Herrlich, Associate Professor of Medicine, Washington University School of Medicine in St. Louis, MO email: aherrlich@wustl.edu

Supplemental Figure Legends:

Supplemental Fig. 1: TPA induced cleavage of endogenous CD44 in the human breast cancer cell line MDA-MB-231. Cleavage is inhibited by batimastat, by the PKC inhibitors BIM1 and Gö6976, by okadaic acid and by an active mutant of merlin (see also[28,29]).

Supplemental Fig. 2: TPA induced increased chymotrypsin sensitivity of endogenous NRG1.

Supplemental Fig. 3: (**A**) NRG1-S286A reduces protease accessibility of the NRG1 ectodomain to chymotrypsin.

(**B**) PKCδ knockdown reduces protease accessibility of the NRG1 ectodomain to chymotrypsin.

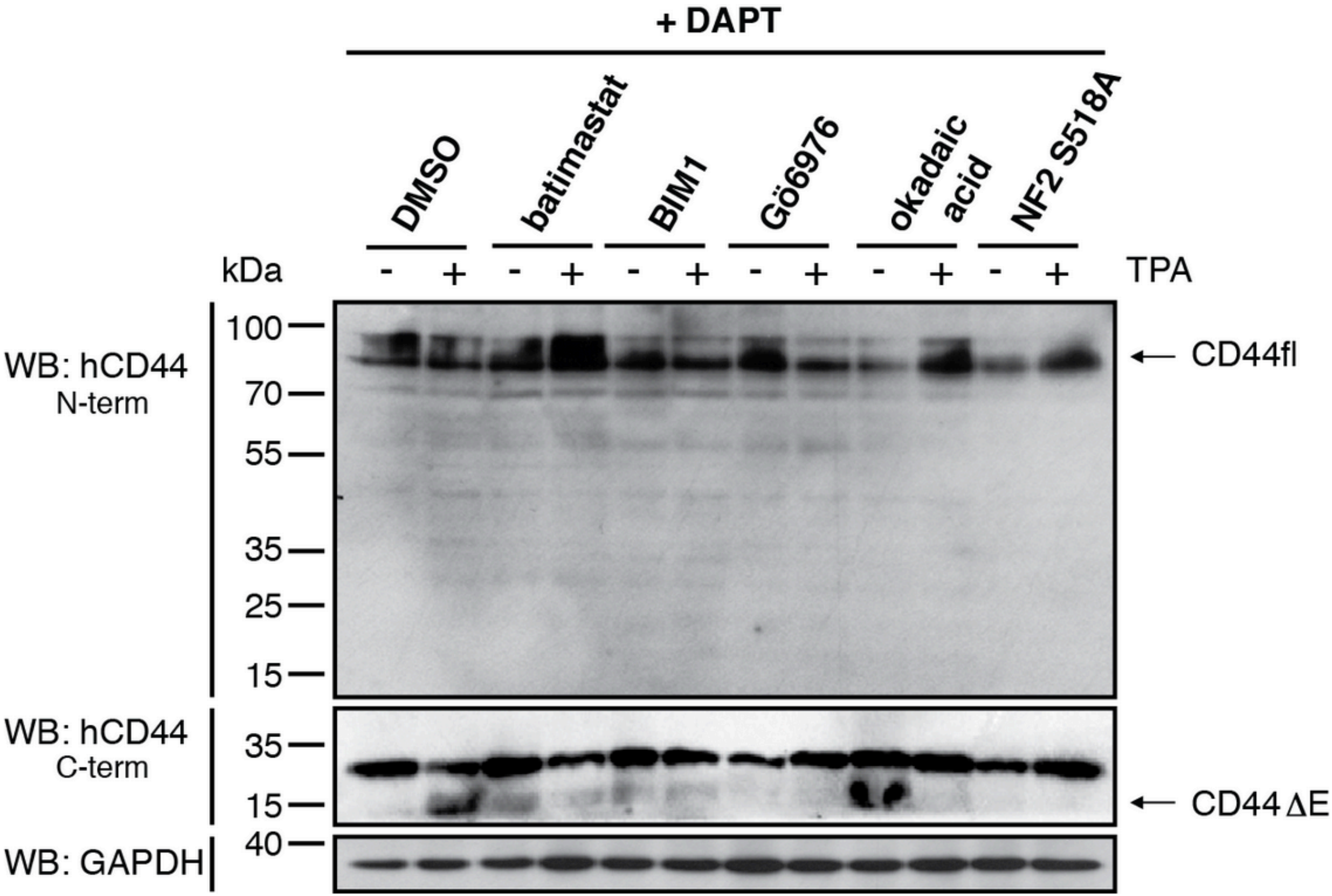

+ DAPT
DMSO
batimastat
BIM1
Gö6976
okadaic acid
NF2 S518A
kDa
- + - + - + - + - + - +
TPA
WB: hCD44
N-term
100
70
55
35
25
15
CD44fl
WB: hCD44
C-term
35
15
CD44 ΔE
WB: GAPDH
40

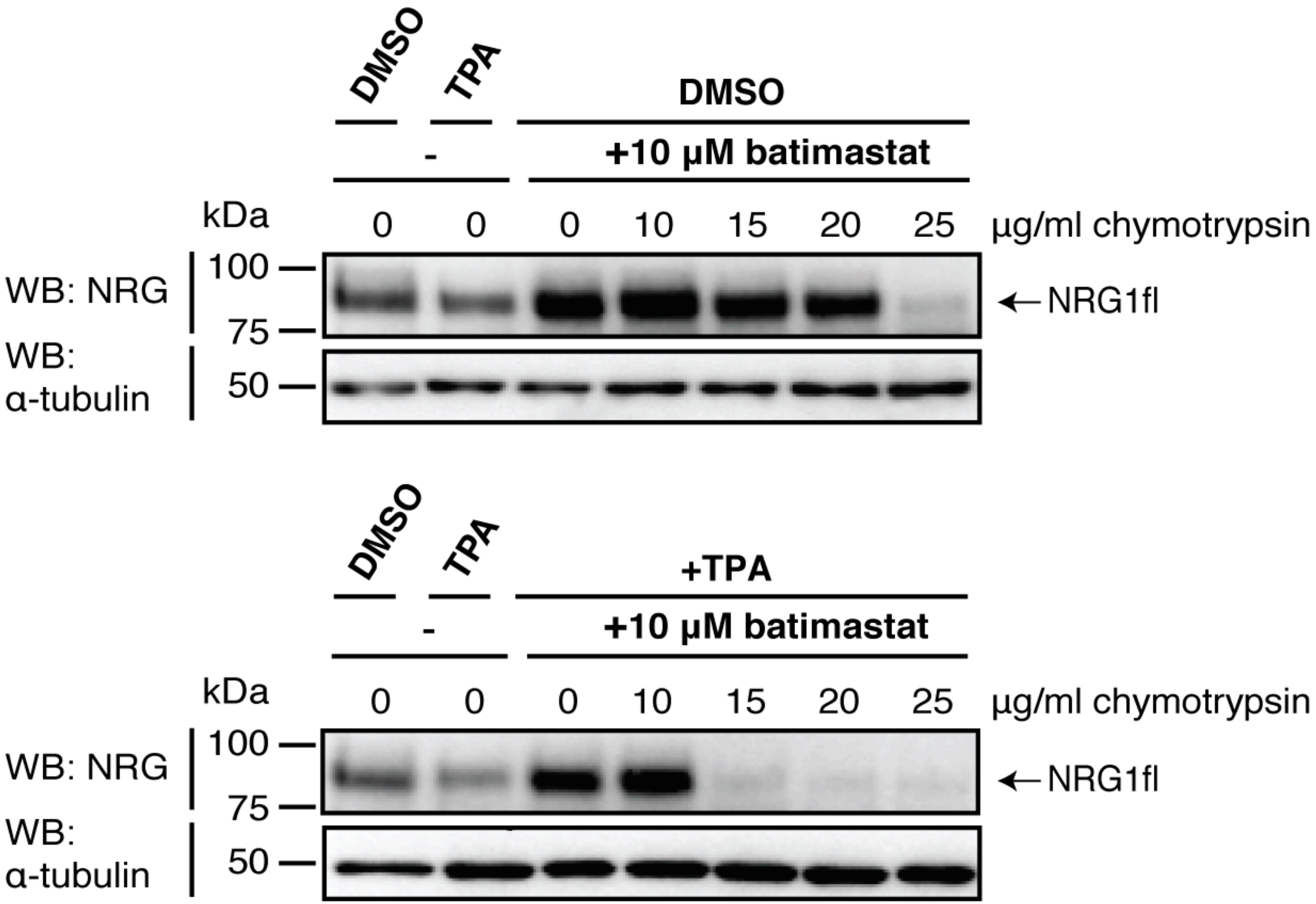

DMSO
TPA
DMSO
-
+10 µM batimastat
kDa
0 0 0 10 15 20 25 µg/ml chymotrypsin
WB: NRG
100
75
NRG1fl
WB: α-tubulin
50
DMSO
TPA
+TPA
-
+10 µM batimastat
kDa
0 0 0 10 15 20 25 µg/ml chymotrypsin
WB: NRG
100
75
NRG1fl
WB: α-tubulin
50

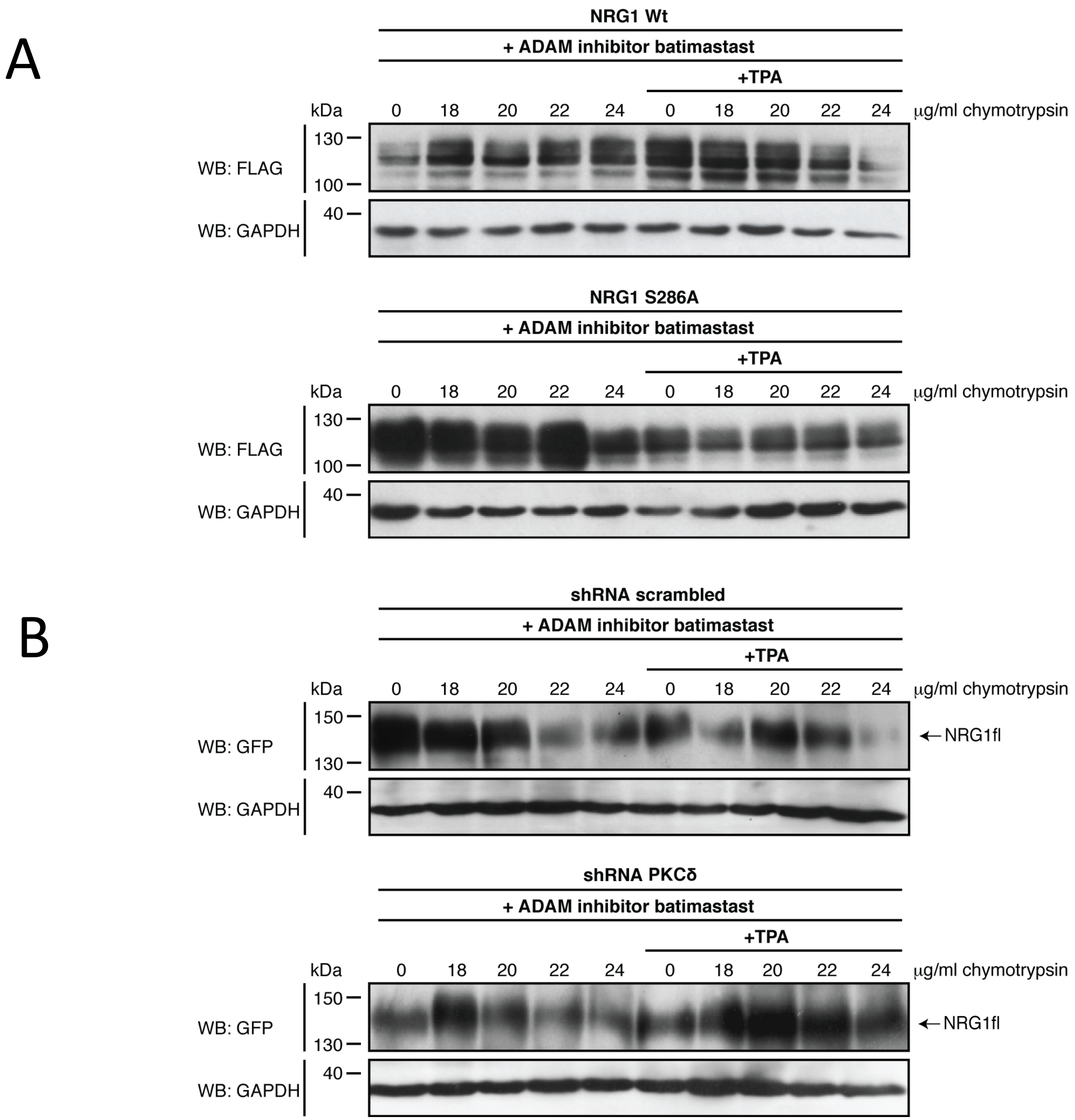
A
NRG1 Wt
+ ADAM inhibitor batimastast
+TPA
kDa
0 18 20 22 24 0 18 20 22 24 μg/ml chymotrypsin
WB: FLAG
130
100
WB: GAPDH
40
NRG1 S286A
+ ADAM inhibitor batimastast
+TPA
kDa
0 18 20 22 24 0 18 20 22 24 μg/ml chymotrypsin
WB: FLAG
130
100
WB: GAPDH
40
B
shRNA scrambled
+ ADAM inhibitor batimastast
+TPA
kDa
0 18 20 22 24 0 18 20 22 24 μg/ml chymotrypsin
WB: GFP
150
130
NRG1fl
WB: GAPDH
40
shRNA PKCδ
+ ADAM inhibitor batimastast
+TPA
kDa
0 18 20 22 24 0 18 20 22 24 μg/ml chymotrypsin
WB: GFP
150
130
NRG1fl
WB: GAPDH
40
